# A new mathematical analysis of randomized trials applied to critical care syndromes: a review

**DOI:** 10.1186/s13037-026-00499-7

**Published:** 2026-07-16

**Authors:** Lawrence Lynn

**Affiliations:** OhioHealth, 1251 Dublin Road, Columbus, OH 43215 USA

**Keywords:** Randomized controlled trials, Critical care syndromes, Transportability, Synthetic data-generating processes, Biological data-generating processes, Cause-agnostic randomized controlled trials, Causal-integrity randomized controlled trials, Structural causal modeling, Treatment-effect estimation, Guideline reversal

## Abstract

Guideline reversals and reversals of randomized controlled trial (RCT) treatment effect estimates have been repeatedly documented in critical care syndrome research over several decades. Critical care syndrome RCTs differ structurally from conventional disease-based RCTs. Rather than enrolling patients on the basis of a specific disease or causal mechanism, they typically employ consensus-derived eligibility criteria composed of disease-and cause-agnostic prognostic physiological thresholds, laboratory abnormalities, and/or nonspecific clinical features. These enrollment criteria select a heterogeneous mixture of distinct diseases and causal systems which are mixed into a single trial. Consequently, the resulting treatment-effect estimate is not the average treatment effect of a single disease or causal mechanism. Instead, it represents a second-order average: an overall average generated by averaging disease or causal system-specific average treatment effects across multiple distinct causal systems. The resulting estimate is therefore best understood as an artefactual “mean of causally unrelated means”: a mixture-weighted average of different treatment effects arising from biologically distinct disease processes. The proposed framework implies that the prevalent structure may produce unstable effect estimates and a “cause–mixture paradox,” in which observed treatment effects reverse to the null or opposite sign across trials as the disease mixture of each trial shifts. This framework comprises a new tool for the mathematical assessment of evidentiary strength of critical care trials under guideline consideration.

## Introduction

Guideline reversals, sometimes associated with harm, and reversals of randomized trial effect estimates have been repeatedly observed in critical care syndrome trials for decades [1]. Substantial methodological effort has focused on understanding heterogeneity of treatment effects, which is commonly invoked to explain these inconsistencies [2–4]. However, the rapid rejection by experienced clinicians [5] of key components of widely promulgated Surviving Sepsis Campaign (SCC) COVID-19 ventilator guidelines [6], themselves derived from highly cited meta-analyses of randomized controlled trials [7], prompted a recent formal failure-mode analysis of critical care syndrome RCTs and their associated guideline reversals [1].

This analysis [1] discovered that a second structural species of randomized controlled trial (RCT) has evolved within critical care medicine, such that two distinct RCT architectures are now in common use, differentiated primarily by how patients are selected through the enrollment criteria (gate) [8]. The purpose of the present review, the third paper in a series of failure-mode investigations of critical care syndrome trials, is to provide a mathematical analysis of syndrome-based randomized controlled trials (RCTs) in response to the hypothesis advanced in two earlier reviews [1, 8]: that the standard trial structure used to study a critical care syndrome generates a treatment-effect estimate whose transportability is often inherently unstable because disease- and cause-agnostic enrollment gates create artefactual disease mixtures whose composition may vary across settings, populations, and time. This causes the resulting treatment-effect estimate to reflect the mixture rather than a coherent biological causal system. A second goal of this review is to provide a framework for guideline developers to assist in the evaluation of the strength of the evidence of clinical trials.

## The diverging species of randomized controlled trials

The ‘estimand’ is a precise description of the ‘treatment effect estimate’ derived from a clinical trial. The estimand framework defines a treatment-effect estimate as a precise description of the effect of interest within a specified population, outcome, and summary measure. However, current guidelines do not explicitly address whether the selected study population represents a coherent biological causal system [9]. The key point is that a treatment-effect estimate cannot be separated from the structure of the trial that generated it, including the enrollment gate. Importantly, consensus acceptance of an enrollment gate does not by itself establish a causally coherent selection mechanism or alter the mathematical properties of the estimand it generates. Under the framework presented here, the concept of the estimand is extended beyond the conventional International Council for Harmonisation of Technical Requirements for Pharmaceuticals for Human Use (ICH) definition [9] to include the mathematical treatment-effect object generated by a trial’s structural design. In this formulation, the enrollment gate is an intrinsic determinant of the estimand because it determines whether the trial estimates a disease- (or causal mechanism-) specific treatment effect or a disease- (or causal mechanism-) mixture treatment effect.

A recent analysis [8] reveals that two different structural sepsis species of RCT are now in wide use in critical care. The original RCT species is the causal-integrity RCT (CIR). Here, the enrollment gate is deliberately constructed to select patients aligned with the specific disease or causal mechanism targeted by the intervention. This design follows the original framework of Ronald Fisher and Austin Bradford Hill [10, 11]. As a result, the effect estimate corresponds to an average treatment effect within a tested set of participants who all have the same treatment targeted causal disease or mechanism so that the average treatment effect reflects variation across covariates, (age, severity, co-morbidity) within a common target causal system [11].

The newer, second RCT species is the cause-agnostic randomized controlled trial (CAR) [8]. The canonical CAR design, using a threshold set as a “triage selection gate”, was standardized in critical care in 1992 [12]. This trial design uses a broad, nonspecific enrollment gate derived from a clinical similarity heuristic. Today, it remains standard practice in critical care science to enroll patients into a RCT on the basis of prognostic thresholds, shared nonspecific radiographic findings, and cause-agnostic physiological or laboratory abnormalities rather than a common disease process or coherent causal mechanism. As a result, eligibility is determined by observable similarities in presentation rather than by demonstrated mechanistic unity [1, 8].

The following framework of “species of treatment effect estimates or estimands” examines the mathematical difference between these two species. It does not challenge the internal validity of randomized controlled trials. Rather, it clarifies that a treatment-effect estimate (estimand) may be internally valid for the constructed mixture-level treatment effect, yet be misinterpreted as a stable, syndrome-specific treatment effect. It is then treated as though it is the evidentiary equivalent of a disease- or mechanism-specific treatment effect and, on that basis, is erroneously considered safely transportable. To make this distinction explicit, the RCT can be conceptualized as a three-layer estimand structure shown in Box 1.

- Layer 1 (E₁): Individual-level treatment effect within a specific disease.
- Layer 2 (E₂): Average treatment effect within a disease or mechanism.
- Layer 3 (E₃): The overall average treatment effect of the average treatment effects of a gate-defined mixture of different diseases or mechanisms. (The mean of causally unrelated means.)

**Box 1:**
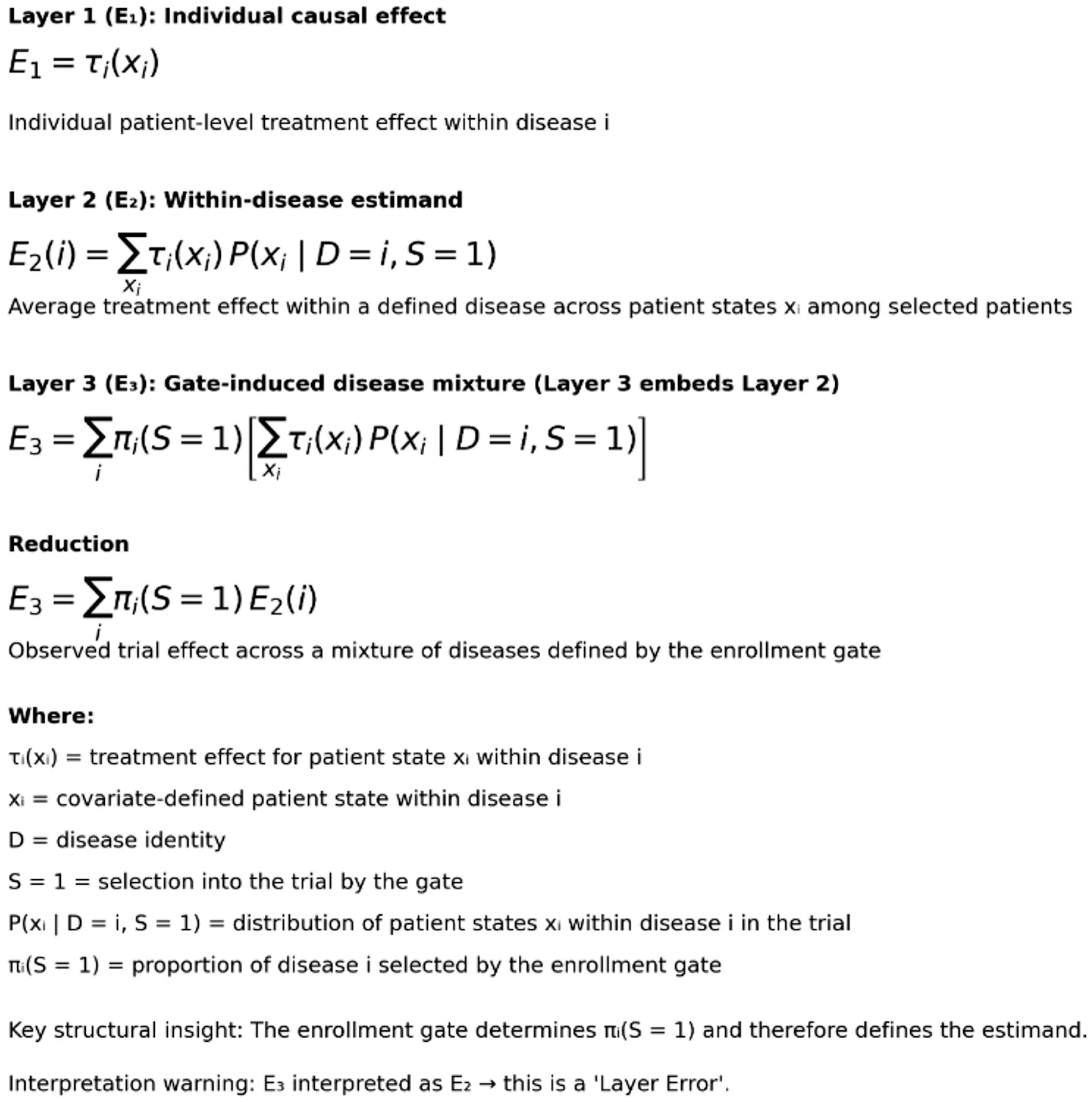
The three-layer trial estimand

## Clinician interpretation of the 2nd and 3rd layers of the three-layer treatment effect estimate (estimand)

$$\:\mathrm{L}\mathrm{a}\mathrm{y}\mathrm{e}\mathrm{r}\:2.\:{\mathrm{E}}_{2}\left(\mathrm{i}\right)={\sum\:}_{\mathrm{x}\mathrm{i}}{\uptau}_{\mathrm{i}}\left({\mathrm{x}}_{\mathrm{i}}\right)\cdot\:\mathrm{P}\left(\mathrm{x}_\mathrm{i}\:\right|\:\mathrm{D}=\mathrm{i},\:\mathrm{S}=1)$$

### Where: the summation is taken over all patient states (covariate profiles) x_i_ within the disease or causal mechanism i

In words: In a causal-integrity RCT (CIR) testing a treatment of a fixed causal mechanism or disease i, the treatment effect τ_i_(x_i_) is evaluated at each patient state x_i_, (e.g., age, severity, comorbidity) weighted by the proportion of selected (trial) patients with that state, and summed across all states to yield the disease-specific average treatment effect E2(i) of the participants in the trial.

$$\:\mathrm{L}\mathrm{a}\mathrm{y}\mathrm{e}\mathrm{r}\:3.\:{\mathrm{E}}_{3}={\sum\:}_{\mathrm{i}}{\uppi}_{\mathrm{i}}(\mathrm{S}=1)\cdot\:{\mathrm{E}}_{2}\left(\mathrm{i}\right)$$

### Where: the summation is taken over all diseases or causal mechanisms selected into the trial population by the enrollment gate (S = 1)

In a cause-agnostic randomized controlled trial (RCT), the selection gate (S = 1) induces a mixture of different causal diseases rather than a single disease or causal mechanism. For each disease i, a disease-specific average treatment effect E2(i) is defined. The trial estimand is then constructed as a mixture-weighted average of these disease-level effects: each E2(i) is weighted by the proportion of that disease among patients selected by the gate, π_i_(S = 1), and summed across all of the diseases in the trial. Thus, the trial estimates a “disease-mixture average treatment effect” of multiple “disease-specific average treatment effects”. Unless the trial is carefully designed, as discussed below, the result is likely an artifact of the mixture and the selection process and not a treatment effect estimate of the perceived syndrome, which is simply a synthetic data generating process (as defined below).

### At Layer 1 (E₁)

The clinician’s question is;

*What is the effect of this treatment in this patient with this disease?*

This is the ideal target of bedside decision-making, but it is not directly observable and not identifiable without additional assumptions beyond the scope of this paper.

### At Layer 2 (E₂)

The cause-integrity RCT question is;

*What is the average effect of this treatment in the trial participants with this disease or mechanism?*

The joint distribution P(x_i_ | D = i, S = 1) describes how patient profiles (e.g., age, severity, comorbidities) vary within the single disease or mechanism studied within the trial.

### At Layer 3 (E₃)

The cause-agnostic RCT question is;

*What is the mixture-weighted average of the disease-specific average treatment effects arising from the distinct diseases or causal systems represented in the participants selected by the enrollment criteria?*

The probability π_i_(S = 1) describes how different diseases are mixed in the trial population. Clinically, π_i_(S = 1) answers: “What percentage of the trial is made up of each disease or causal mechanism?” These mixture weights determine how disease-specific effects are combined to produce the overall trial result.

- P describes how patients vary (e.g., age, severity, comorbidity) within a single disease RCT (Layer 2).
- π describes how different diseases are mixed by the selection gate within a syndrome RCT (Layer 3).

Maintaining this distinction is essential for guideline development. An “Estimand Layer Error” occurs when a disease mixture-level (Layer 3) estimate in interpreted as if it is the evidence level equivalent of disease-specific (Layer 2) effect. This can inflate the perceived “level of evidence” and lead to inappropriate clinical recommendations. This form of misattribution contributed to difficulties in, and the ultimate rejection of, the act of applying ARDS trial results to COVID-19 ventilator guidelines [5, 13–15]. Table 1 contrasts the CIR and CAR generated estimand.

**Table 1:** P vs. π: Covariate joint distribution vs. disease mixture percentages

Feature	*P*(x_i_ | D = i, S = 1)	π_i_(S = 1)
What it is	Joint probability distribution	Probability (mixture weight)
What varies	Covariate vector x_i_ (patient profiles)	Disease
Level	Within a single disease	Across different diseases
Causal structure	One causal system	Multiple causal systems
Role in estimand	Weights patient profiles in Layer 2	Weights diseases in layer 3
Clinical meaning	“What % of patients with this disease look like this?”	“What % of the trial is each disease?”
Type of variation	Heterogeneity of treatment effects (within disease)	Mixture (between diseases)
Mathematical object	Distribution over many covariate values	Set of probabilities across diseases
What gets averaged	Patient-Disease level effects	Disease mixture level effects

Contrast the estimand at Layer 2 with that of Layer 3. Note that these are layers. So the complexity of the second (single disease or mechanism) layer is carried into the third for each disease included in the mixture. Contrast this Table with Fig. 2 where the Layer 2 DAG on the left and the Layer 3 DAG on the right correspond to the left and right panels of this Table

## The clinical question that redefined the trial treatment effect estimate

Austin Bradford Hill described the clinician’s question as fundamentally causal: what is the effect of this treatment for this disease in this patient? When anchored to a specific disease, this corresponds to Layer 1 (E₁), the individual-level treatment effect, which is not directly observable even in a causally anchored randomized trial; such trials instead estimate the average treatment effect at Layer 2 (E₂). In causal-integrity randomized controlled trials (CIRs), enrollment is aligned to a specific disease or mechanism, preserving this disease or mechanism specific estimand.

In contrast, modern critical care trials often address a different question: what is the effect of treatment in a syndrome defined by shared prognostic markers and shared clinical features but composed of groups of patients with different, often unidentified, underlying diseases which produce this same basic presentation? This question cannot be answered at Bradford Hill’s Layer 2, which is inherently disease or causal-mechanism specific.

To address this, the cause-agnostic randomized controlled trial (CAR), a distinctly new species of RCT structure was developed for the study of the sepsis syndrome in 1987 [16]. This seemed, at the time, a natural process, after all, a disease is diagnosed by disease specific abnormal physiological values so the idea developed that a syndrome could be defined by consensus criteria comprised of abnormal physiological values. To trialists and statisticians these seemed like equivalent RCT gates.

**Fig. 1:**
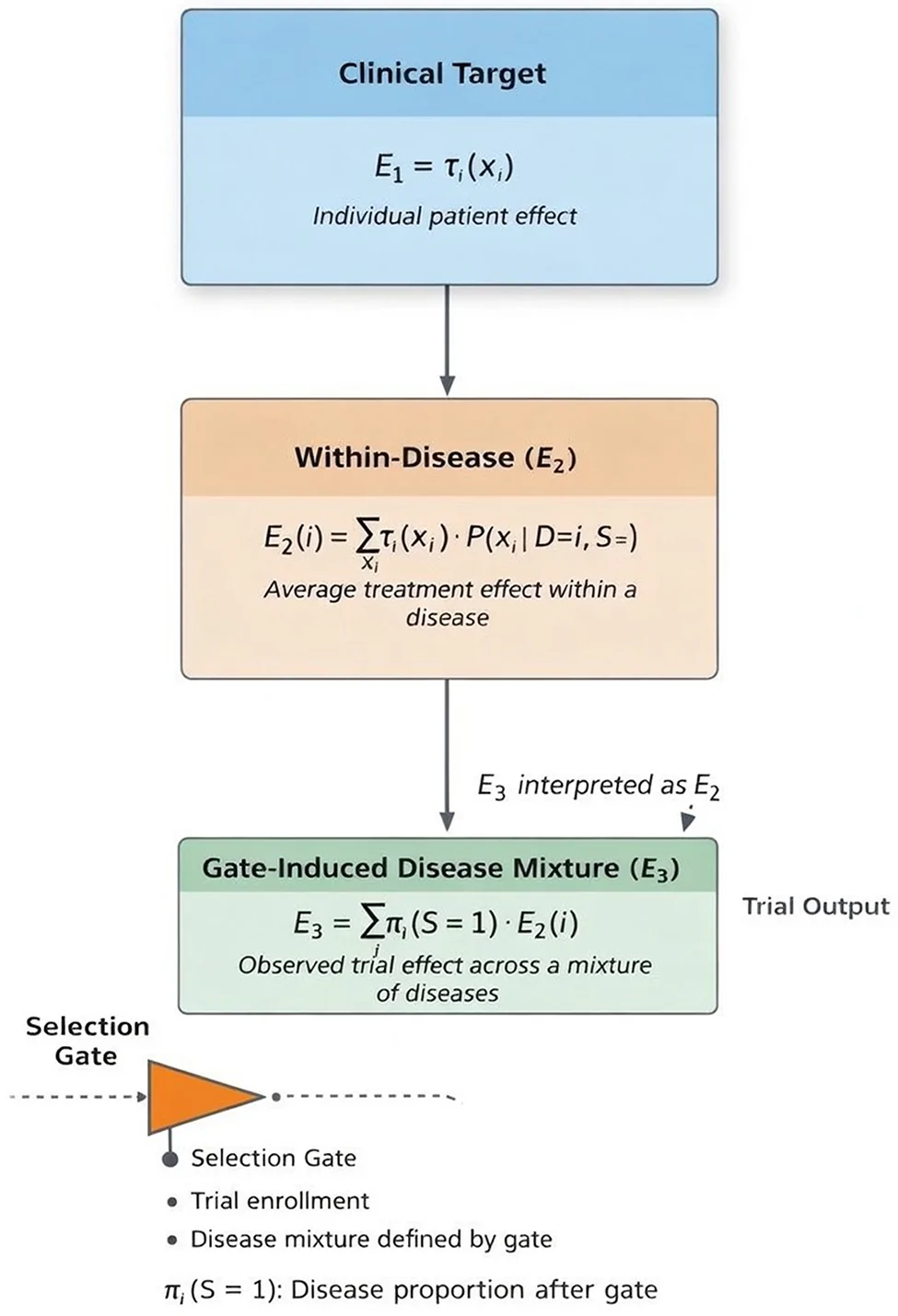
The three-layer trial estimand. Depicts the three-layer structure of treatment effect estimation in randomized controlled trials. The arrow labeled “selection gate” emphasizes that the enrollment process constructs the mixture by determining which diseases, and in what proportions, enter the trial. As a result, E₃ reflects a gate-defined mixture effect rather than a disease-specific effect. A key interpretive risk is indicated by the arrow labeled “E₃ interpreted as E₂.” This represents a “estimand layer-error”, defined as the misinterpretation of a treatment effect estimated at one causal layer (e.g., a gate-defined mixture, E₃) as if it provides an equivalent “level of evidence” as a causal coherent layer (e.g., a disease-specific effect, E₂)

However, in this new RCT species (the CAR), the treatment effect estimate (estimand) shifted to a new layer never anticipated by Fisher or Bradford Hill; Layer 3 (E_3_) as depicted in Fig. 1. From a causal inference perspective, this is a transportability problem since the treatment effect estimate depends on how the study population is constructed. In this new alternative design, the gate no longer functions as a disease or causal mechanism classifier, but as a system that uses consensus derived disease agnostic physiologic threshold as a “triage selection gate” to generate a mixture of underlying diseases for inclusion in the trial. While this seemed reasonable at the time, this was a radical change in trial architecture.

**Fig. 2:**
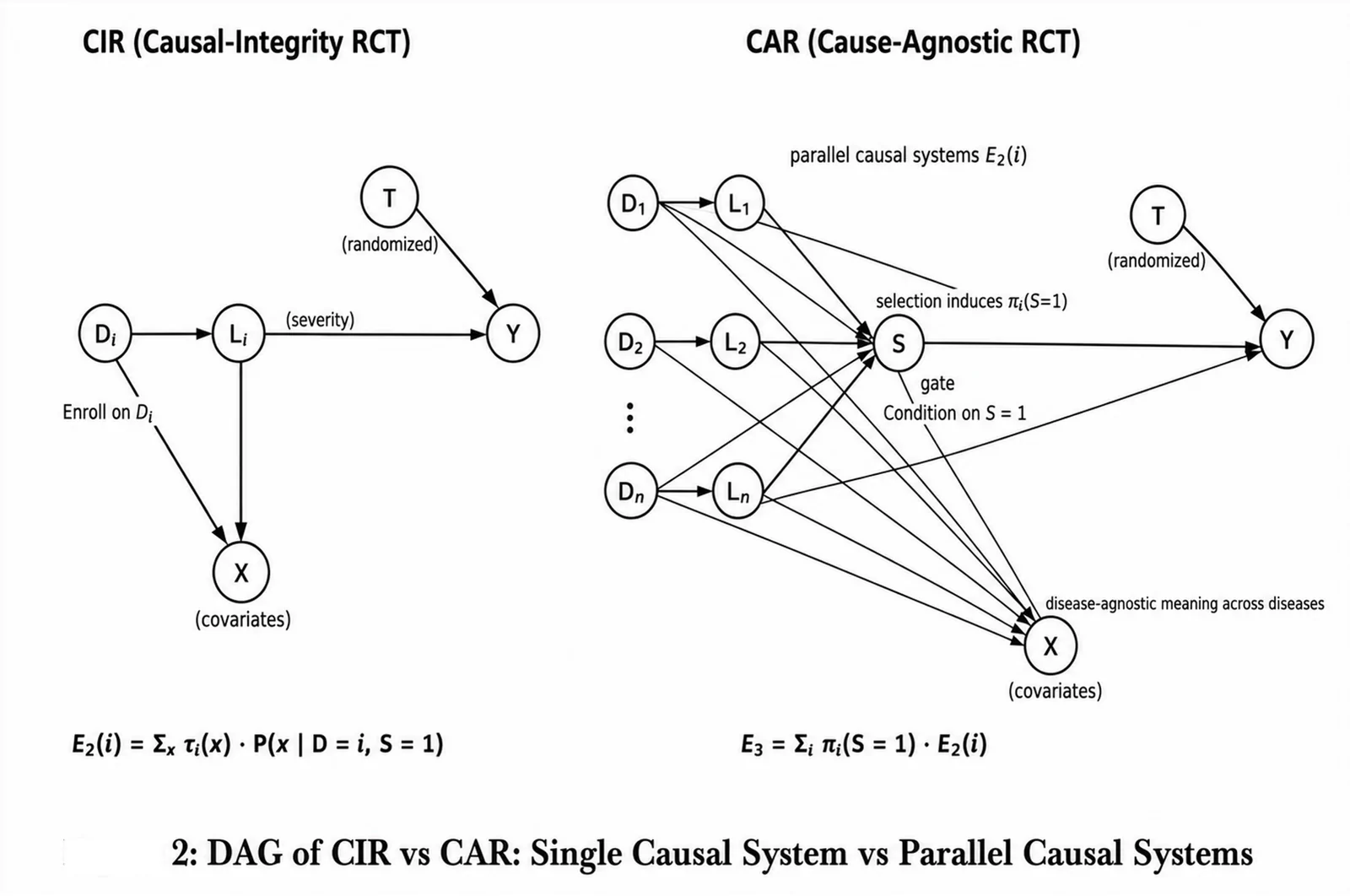
DAG of CIR vs. CAR: single causal system vs. parallel causal systems. Shows the expansive effect of adding this third layer. This is visualized by comparing the directed acyclic graphs (DAGs) of the cause integrity RCT (equivalent to Layer 2) (on left) with the cause-agnostic RCT (CAR) which adds layer 3. Here the structural expansion of the heterogeneity is evident by the parallel-causal- systems and this is further complicated by the dissociation of the covariate vector from any common causal pathway. Although not shown to reduce complexity of the drawing, covariates may also have a path to the gate (S = 1) when disease and mechanism agnostic physiologic thresholds are used for selection

The effect of the layers on the causal structure of a cause-agnostic RCT is shown in Fig. 2.

## The “cause-mixture paradox”

A “cause mixture paradox” can arise in sequences of cause-agnostic RCTs. In these trials, each disease may have a consistent treatment effect, but the overall result depends on the proportion of diseases included.

In a toy example, presented in Table 2, suppose the consensus group defines “Syndrome X” which includes two unknown diseases and then tests treatment for this syndrome. If Disease A has a positive effect and Disease B has a negative effect, the overall outcome changes as the disease proportions shift. In one trial, with a higher proportion of Disease A yields a positive net effect for the treatment of syndrome X; in another trial, with a higher proportion of Disease B yields a negative effect for the identical treatment of syndrome X. Thus, even with stable disease-level effects, sequential trials can produce different conclusions.

This paradox is a structural consequence of cause-agnostic design: the estimand reflects a changing mixture, not a stable effect for a single condition. Of course syndromes often have very many different diseases sometimes more than 10 which are captured by the gate, so the shift of the treatment effect is unpredictable.

**Table 2:** The cause-mixture paradox

Scenario	π_i_ (Disease A)	π₂ (Disease B)	E₂(A)	E₂(B)	E₃ = Σ_i_ π_i_ (S = 1) ·E₂(i)	Interpretation
Trial 1	0.80	0.20	+0.20	−0.20	+0.12	Treatment first appears beneficial
Trial 2	0.20	0.80	+0.20	−0.20	−0.12	Treatment now appears harmful

Shows a toy example revealing a cause mixture paradox

Here we consider two underlying diseases contributing to a syndrome-defined trial population. Let Disease A have a positive treatment effect (E₂(A) = + 0.20) and Disease B a negative effect (E₂(B) = − 0.20). If the disease-and cause-agnostic enrollment gate selects a group of participant with π_i_ (percentage) = 0.80 and π₂ (percentage) = 0.20, then E₃ = +0.12. If a second trial using the identical syndrome definition but differing in disease selection, yields π_i_ = 0.20 and π₂ = 0.80, then E₃ = −0.12. Thus, identical perceived “syndrome-specific effects” can produce opposite trial-level conclusions solely due to gate-induced mixture shifts despite the same enrollment criteria

Key point: Neither RCT estimates a transportable “syndrome treatment effect”, each trial estimated a non-reproducible and non-transportable weighted average defined by its own enrollment gate

$$\begin{aligned}&\mathrm{O}\mathrm{b}\mathrm{s}\mathrm{e}\mathrm{r}\mathrm{v}\mathrm{e}\mathrm{d}\:\left(\mathrm{t}\mathrm{r}\mathrm{a}\mathrm{n}\mathrm{s}\mathrm{p}\mathrm{o}\mathrm{r}\mathrm{t}\mathrm{e}\mathrm{d}\right)\:\mathrm{t}\mathrm{r}\mathrm{i}\mathrm{a}\mathrm{l}\:\mathrm{e}\mathrm{s}\mathrm{t}\mathrm{i}\mathrm{m}\mathrm{a}\mathrm{n}\mathrm{d}\:{\mathrm{E}}_{3}\cr&\quad={\sum\:}_{\mathrm{i}}{\uppi}_{\mathrm{i}}(\mathrm{S}=1)\cdot\:{\mathrm{E}}_{2}\left(\mathrm{i}\right)\end{aligned}$$

This differs from Simpson’s paradox, where a trend in separate groups reverses when combined, often due to confounding [17]. Simpson’s paradox is a statistical artifact that can often be addressed through appropriate adjustment. In contrast, the cause-mixture paradox arises from variation in the underlying composition of causal systems across trials and cannot be resolved through statistical adjustment alone, as it reflects a shift in the estimand itself.

##  Heterogeneity of treatment effects vs. cause-mixture heterogeneity

Heterogeneity of treatment effects (HTE), is the variation in the magnitude or direction of a treatment effect across individuals or patient subgroups within a common disease or causal mechanism, typically attributable to differences in patient characteristics (covariates), disease severity, genetics, or other effect modifiers. Contemporary approaches to heterogeneity of treatment effects generally model variation in treatment response within a pre-specified study population as a function of covariates and their interactions [2, 18–20]. Implicit in this approach is the assumption that the study population can be represented within a common covariate space in which variables retain a stable interpretation across participants. The possibility that the enrollment gate may combine multiple distinct biological causal systems is generally not the primary focus of these methods.

However, in cause-agnostic trials, this assumption may not hold. The enrolled population may represent a mixture of parallel causal systems, each with its own covariate distribution and treatment response function. In this setting, observed heterogeneity reflects not only the within- system variation but also between-system differences induced by the enrollment gate.

Because each disease or causal system is associated with its own covariate structure, the observed covariates are drawn from a mixture of disease-specific distributions rather than a single coherent space. The same observed variable may therefore encode different roles across systems, for example, a physiologic measurement may represent disease severity in one condition, compensation in another, and be unrelated in a third.

This limitation becomes clearer when heterogeneity is viewed through the three-layer estimand framework. Classical heterogeneity of treatment effects is well-defined within the coherent disease mechanism represented at E₂. Cause-agnostic trials (CARs) operate at a different layer. The cause mixture associated heterogeneity in these trials is not classical heterogeneity of treatment effects made more complex; it is structurally different. The triage selection gate (S) of the CAR, together with the available population to which the gate is applied, determines which diseases or causal mechanisms enter the trial, how many are represented, and their relative proportions. In doing so, it constructs the potentially artefactual third-layer estimand itself. This distinction is fundamental and is illustrated by the comparison DAGs in Fig. 2.

## When cause-agnostic gated trials work: pathophysiology as a unifying mechanism

Cause-agnostic and causal-integrity RCTs lie along a continuum. The dichotomous taxonomy is presented primarily as a pedagogical framework [8]. Historically, the original investigators who streamlined Bradford Hill’s original disease-linked trial logic into broad cause-agnostic RCT of synthetic syndromes did not believe they were creating a new species of trial. They believed that the syndromic defining thresholds (RCT gate) captured a shared physiologic state. Thomas Petty, for example, believed that his hypothesized syndrome of ARDS was caused by surfactant depletion across a wide range of diverse pulmonary diseases. If heterogeneous diseases truly converge on a common causal pathway, then aggregation may be justified, as disease-specific treatment effects collapse onto a pathway-level effect. When this is true, disease mixture collapses toward mechanism and the estimand begins to move back toward E₂ and the RCT is not cause-agnostic. However, this condition is rarely satisfied in practice.

This aggregation is valid only when the physiologic variable collapses the mixture at the level of causal mechanism, reflecting a shared disturbance, causally linked to outcome, serving as a valid measure of the underlying pathway, and directly modifiable by the intervention. Otherwise, it creates a new synthetic layer under a physiologic phenotype label rather than resolving the original problem.

One example of genuine physiologic disease unification is the low inflatable alveolar volume of the “baby lung.” Across distinct causes of alveolar filling or collapse, a shared vulnerability to ventilator-induced lung injury emerges. This concept underlies the success of lung-protective ventilation strategies demonstrated in randomized trials [21]. This shared causal target represents a post gate physiologic funnel rendering a more causally coherent enrollment than a broad syndrome gate by itself. However, even here, the threshold gate remains a hazard if it does not reliably capture the targeted low volume physiologic state. Broader criteria based on PaO2/FIO2 may select patients who do not actually share low inflatable alveolar volume and therefore may not share the relevant causal vulnerability. If the ARDS criteria are so broadened by a consensus panel without these consideration, future RCT estimates might reverse and find no benefit to lung protective ventilation.

## Low PaO₂/FIO₂ as a pseudo-unifying target

PaO₂/FIO₂ is often treated as though it defines a unifying physiologic state across diverse diseases, useful as a gate for trials. The problem is that unlike the straight forward low volume “baby lung’ example, a low PaO₂/FIO2 is not a disease unifying mechanism. It is a composite signal generated by several highly relational time dimensioned processes. A useful way to see this is with the classical shunt equation:

$$\:\mathrm{Q}\mathrm{s}/\mathrm{Q}\mathrm{t}=({\mathrm{C}\mathrm{c}\mathrm{O}}_{2}-{\mathrm{C}\mathrm{c}\mathrm{O}}_{2})/({\mathrm{C}\mathrm{c}\mathrm{O}}_{2}-{\mathrm{C}\mathrm{v}\mathrm{O}}_{2})$$

This reflects well-established respiratory physiology wherein PaO2/FIO2 depends not only on alveolar function but also on perfusion distribution, mixed venous oxygen content, and cardiac output [22, 23]. In words, the measured degree of shunt (Qs/Qt) depends on the oxygen content of end-pulmonary capillary blood (CcO₂), arterial blood (CaO₂), and mixed venous blood (CvO₂). Mixed venous oxygen content is itself responsive to oxygen consumption, peripheral shunting, and cardiac output. Thus, PaO2/FIO2 reflects not only alveolar recruitment but also, the lung perfusion distribution, oxygen extraction, cardiac output, and mixed venous oxygen saturation.

A single PaO₂/FIO₂ value can therefore arise from very different relational physiologies. One patient may have recruitable edema with preserved cardiac output. Another may have a very low mixed venous oxygen with a permissive shunt. Another may have fixed consolidation with little recruitability. Rather than collapsing heterogeneity, PaO₂/FIO₂ can conceal it and thereby introduce a new synthetic layer.

The distortion deepens when the same variable is used both to select patients and to guide treatment with positive end expiratory pressure (PEEP). As a result, the pooled estimate averages across heterogeneous physiologic control loops, not merely across patient populations.

The problem is that PEEP does not simply improve PaO₂; it alters the determinants of PaO₂ sometimes in a harmful way. It can recruit alveoli, redistribute blood flow, alter shunt fraction, reduce venous return, lower cardiac output, and thereby reduce the mixed venous oxygen content and regional oxygen delivery. The intervention modifies the very signal used to guide the intervention.

**Fig. 3:**
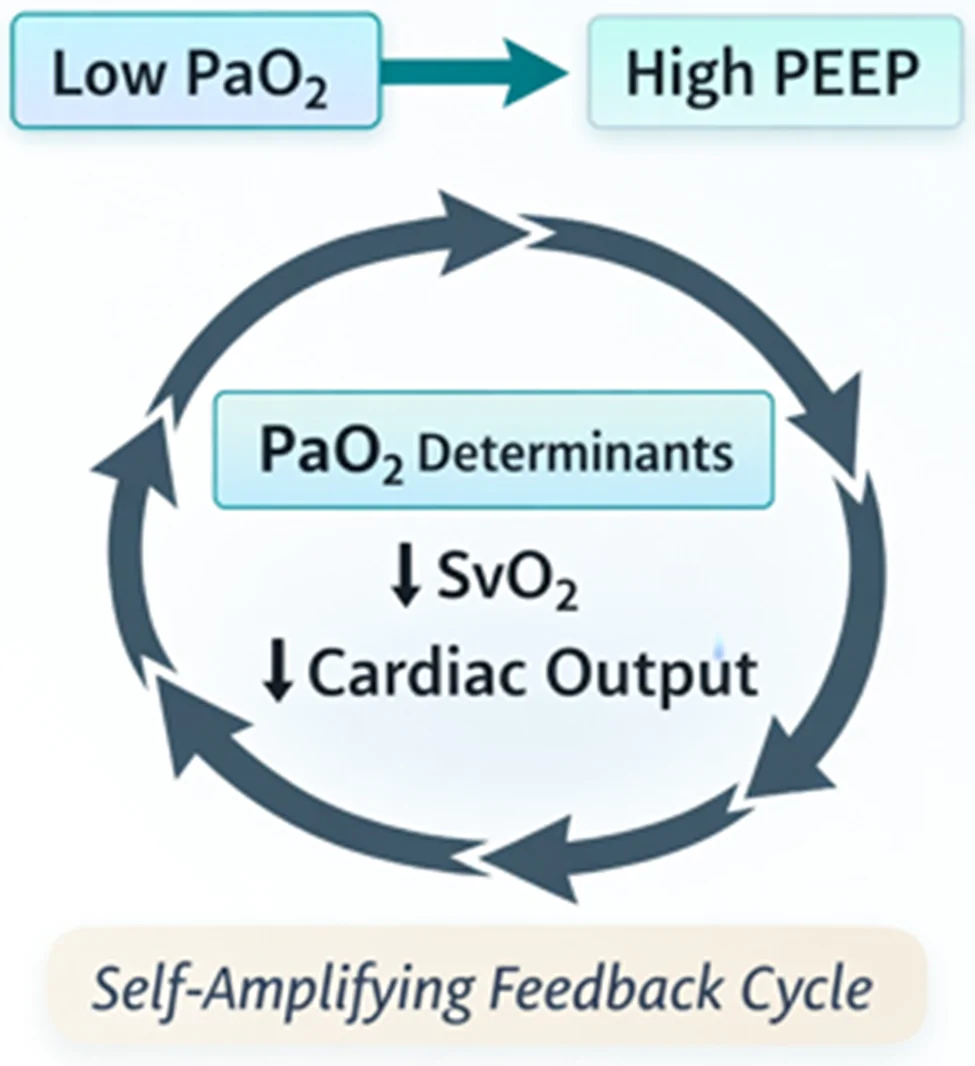
Autopoietic distortion loop. Illustrates a potential adverse PEEP titration loop in a patient vulnerable to PEEP-induced reductions in cardiac output. Increasing PEEP lowers cardiac output, which reduces mixed venous oxygen saturation (SvO₂). In the presence of intrapulmonary shunt, this fall in SvO₂ attenuates the expected rise in arterial oxygen tension (PaO₂), potentially prompting further upward titration of PEEP. The result is a self-reinforcing, deleterious feedback loop

For example, PEEP may increase the PaO2 by reducing the Qs/Qt but at the same time drive cardiac output downward thereby reducing the SVO2 which attenuates the rise in PaO2 which can be misinterpreted as alveolar recruitment failure and this may result in further upward titration in a deleterious cycle (Fig. 3). These relational states do not respond uniformly to PEEP titration. In such cases, the RCT does not measure the treatment effect on a stable causal target. Instead, it measures outcomes in a composite variable whose meaning shifts as the intervention modifies it. In this scenario, a physiologic target does not resolve the disease mixture; rather, it reproduces the heterogeneity in a dynamic form.

## Capillary refill time as conditional unifying target

Unlike a low PaO₂/FIO₂ in diseases of alveolar filling and collapse, a high capillary refill time (CRT) during shock may represent a more straightforward physiologic connection to a unifying treatment target [24–26]. In shock, low tissue perfusion comprises a downstream pathway comprised of organ hypo perfusion, organ failure, and death. For this reason, heterogeneous diseases associated with shock states may converge on a more coherent unifying target.

The ANDROMEDA-SHOCK-2 [26] and earlier such trials represents a transitional design away from the original fully the cause-agnostic RCT design to a cause-agnostic trial that implicitly recognizes the failure of uniform treatment within a syndrome and moves toward causal-integrity principles by introducing physiology-guided treatment alignment. Shifting a trial to the left along the E2–E3 spectrum requires designing it so that treatment assignment and interpretation are less dependent on a broad, cause-agnostic syndrome gate and more closely aligned with a coherent causal mechanism. Tools and methods which guide a trial design to assure it has a favorable position along the E2-E3 spectrum are sorely needed.

For example, CRT would be particularly valuable if it functions to shift the estimand in shock trials from E₃ toward an E₂ equivalent by collapsing disease heterogeneity onto a shared, outcome-relevant disturbance in tissue perfusion. However, like a low PaO2/FIO, a high CRT can arise from multiple, physiologically distinct states. States with high CRT include low cardiac output, impaired venous return, increased vasomotor tone with elevation of critical closing pressure [27], regional maldistribution of flow, or preexisting microcirculatory dysfunction. Accordingly, the intervention required to normalize CRT is contingent on the underlying mechanism driving its prolongation.

By contrast, if elevated CRT were treated using a fixed, protocolized “inotrope titration table” (analogous to PEEP–FIO₂ tables used for hypoxemia in ARDS trials), the heterogeneity of these underlying mechanisms would be obscured. In such a framework, CRT would function as a unifying but non-specific target, and its apparent clinical coherence would likely dissolve, much as PaO₂/FIO₂ does when used as a titration endpoint across physiologically diverse states.

For this reason, any candidate physiologic unifier must be empirically characterized as a time-series signal rather than a static threshold. The central question is not whether a value is abnormal, but how it behaves in relation to other signals over time in response to perturbation and recovery within specific shock states? Which forms of shock produce coherent, reproducible trajectories? In which settings does the signal change early, preceding overt clinical deterioration, and in which does it remain noisy, delayed, or discordant with underlying perfusion failure?

These relational times series patterns can be initially defined through retrospective time-series analysis of archived physiologic data in electronic medical records and integrated patient monitor databases.

### Why Bayesian and adaptive methods do not resolve the problem

One approach sometimes applied to cause-agnostic trials involves Bayesian methods or adaptive platform designs that incorporate updating, hierarchical borrowing, or subgroup learning [28, 29]. These methods can indeed sharpen estimates and adaptively allocate based on emerging data. They do not, however, change the causal level of the estimand. More specifically, these methods refine estimation within the defined estimand but do not alter the causal structure of the estimand itself. A more precise estimate of a mixture remains a mixture-level estimand.

For example, in a recent multicenter cause-agnostic (Layer 3) trial of corticosteroids for severe community-acquired pneumonia (CAP), the REMAP-CAP trial [30], the observed treatment effect reversed direction relative to the benefit reported in the guideline-defining CAPE COD trial [31]. Despite the use of advanced statistical methods The REMAP-CAP corticosteroid domain trial was stopped for futility, with no evidence of benefit and a directional signal toward higher mortality in the hydrocortisone group (15% vs. 9.8%; adjusted OR ~ 1.5–1.6, with all credible intervals crossing 1). Despite a point estimate favoring harm, the trial is reported as ‘futile’ because the estimand was framed around probability of benefit.

This constitutes a marked reversal relative to current guideline recommendations supporting corticosteroids in severe CAP [32], and is more consistent with the 2019 guidelines [33], which stated the opposite “*We suggest****not****routinely using corticosteroids in adults with severe CAP (conditional recommendation*,* moderate quality of evidence)*” (emphasis added). These are just the latest of such trials. Numerous trials of corticosteroids in CAP have produced discordant results and these oscillating guideline shifts are consistent with reversing effects predicted by the phenomenon we introduced here as, the cause-mixture paradox of sequential cause-agnostic RCTs.

Examining the layer 3 estimand, E_3_ = Σ_i_ π_i_(S = 1) · E₂(i) we see that the enrollment gate (S = 1) allows any π_i_ (percentage) of any of the CAP infections to pass through into the trial, potentially producing reversal of the aggregate effect despite stable individual disease-specific effects E₂(i). Here S = 1 can select from a biological heterogeneity embedded within community-acquired pneumonia (CAP) which is not a matter of degree but of kind. The CAP label aggregates infections caused by organisms with fundamentally different biological structures, including encapsulated bacteria (e.g., *Streptococcus pneumoniae*), cell wall–deficient bacteria, viruses (e.g., influenza A), and fungi (e.g., *Pneumocystis jirovecii*), spanning multiple biological domains, including many organisms outside the classical domain framework.

These entities differ fundamentally in structure, replication, host interaction, immune response, treatment susceptibility and anti-biologic safety net availability and efficacy (1). To treat such a collection as a single disease entity in an RCT is therefore not merely a simplification but a categorical conflation of distinct causal systems. Under these conditions, a single intervention may plausibly produce benefit in one disease, no effect in another, and harm in a third, rendering the aggregate treatment effect an unpredictable function of the underlying mixture rather than a property of any coherent syndrome. Such a design carries the risk of exhibiting the cause-mixture paradox and unsafe guideline development. Table 3 shows the reversals of CAP guidelines over the past 8 years (although corticosteroid treatment for CAP trials go back more than 20 years).

All CAP RCTs studying corticosteroids as treatment are inherently cause-agnostic, enrolling heterogeneous disease processes under a single syndromic label. In this setting, reversal of the observed treatment effect is not surprising. The effect estimates from both CAPE COD and REMAP-CAP are defined by the enrollment gate and the resulting disease mixture. The CAP example is presented here as an illustrative application of the framework rather than proof of the cause-mixture paradox. However, Table 3 shows a potential example of the cause-mixture paradox with sequential CAP cause-agnostic RCTs.

**Table 3:** Potential example of the cause-mixture paradox manifested in clinical guidelines for severe CAP relying on layer-3 estimands

Source	Year	Population (CAP framework)	Corticosteroid Guideline Recommendation	Implied Estimand	Direction of Effect
Infectious Diseases Society of America / American Thoracic Society CAP Guideline	**2019**	Non-severe + Severe CAP	**against** corticosteroids	E₃ = Σ π_i_(S = 1) · E₂(i)	Inconclusive some trials show benefit/ some harm
American Thoracic Society CAP Guideline Update	**2025** *(current in 2026)*	Severe CAP	**for** corticosteroids	E₃ = Σ π_i_(S = 1) · E₂(i)	Net benefit
REMAP-CAP (platform RCT, steroid domain)	**2025**	Severe CAP (heterogeneous etiologies)	No guideline effect Trial stopped early for futility/harm	E₃ = Σ π_i_(S = 1) · E₂(i)	No benefit /Possible harm

The directionally discordant corticosteroid signals across guidelines and trials, (2019) recommend against, then benefit (2025), and then a strong signal for harm (REMAP-CAP), occur within a syndrome-defined population (CAP) but are consistent with variation in the underlying disease mixture (π_i_). This pattern reflects instability of a layer-3 estimand (E₃), rather than true inconsistency of disease-specific treatment effects (E₂(i))

The issue becomes even more consequential in adaptive platform trials, where the disease mixture may change over time. As the mixture weights become time-dependent, observed changes in treatment effect may reflect changes in the enrolled disease mixture rather than changes in the underlying causal effect. If an early disease mixture favors the intervention, response-adaptive allocation may increase assignment to that intervention. Should the disease mixture subsequently shift toward diseases in which the intervention is ineffective or harmful, the adaptive allocation algorithm may expose a larger proportion of patients to that intervention than would occur under fixed randomization, thereby increasing risk to the trial population. In this circumstance, the platform adapts to an apparent treatment effect generated by a changing disease mixture rather than by identification of the underlying disease-specific causal mechanism.

This is not a limitation of the statistical method rather it reflects the causal structure defined by the use of a triage selection gate.

## Synthetic data-generating processes created by trial design

A “data-generating process” (DGP) refers to the underlying stochastic mechanism that produces observed data. This manuscript introduces the term “biologic DGP” (BDGP) as the underlying biological causal system that generates data and outcomes under specified interventions. In contrast, a gate composed of disease- and cause-agnostic thresholds produces a “synthetic data-generating process” (SDGP) which is essentially created by a consensus panel and trial design.

- Statistical DGP: the stochastic process generating the observed data.
- Biological DGP: the actual disease mechanism or causal system generating the observations.
- Synthetic Data-Generating Process (SDGP): a trial-defined data-generating process that does not correspond to a single biological causal system but instead arises from the aggregation of multiple biological data-generating processes through a disease- and cause-agnostic selection gate. An SDGP is created by the enrollment gate rather than discovered in nature.

In critical care a SDGP is generally perceived to be a causal object, generally a “syndrome”, and it looks very similar to a BDPG until the structure of the RCT is examined closely as by structural causal modeling. The study of SDGPs as causal objects dates back, at least, to 1987 [16]. Here, Roger Bone’s 1987 methylprednisolone trial used a sepsis syndrome triage selection gate based on physiologic abnormalities. This approach was subsequently formalized as the SIRS consensus criteria in 1992 [12]. Here the consensus panel promulgated a sepsis syndrome triage selection gate called the SIRS (the systemic inflammatory response syndrome) criteria which was later replaced by Sepsis 3 [34] based on SOFA (sequential organ failure score) [35]. The SIRS and Sepsis 3 criteria each comprise of a set of prognostic but disease agnostic laboratory and/or physiological thresholds. The SIRS criteria and Sepsis 3 are not themselves SDGPs. Rather, they function as a disease- and cause-agnostic selection gate that generates SDGPs by triage-induced combination of multiple underlying biological data-generating processes into a single set of trial participants. The disease mixture aggregation which results from applying SIRS or Sepsis 3 as a triage selection gate is the SDGP which is studied in the trial. Here it can be readily seen that even using the same gate, each trial tests treatment a new SDGP comprised of a different mix of BDGPs (diseases or causal mechanisms).

In principle, a virtually unlimited number of such trials can be performed since new SDGPs, as also defined by newly created consensus based triage selection gates, can be produced at will. Table 4 provides a comparison of biologic data generating processes (BDGP) and synthetic data generating processes (SDGP).

**Table 4:** Comparison of biological data-generating processes (BDGPs) and synthetic data-generating processes (SDGPs)

Feature	Biological Data-Generating Process (BDGP)	Synthetic Data-Generating Process (SDGP)
Definition	A coherent biological causal system generating observations and outcomes under intervention.	A mixture of diseases and biological causal systems created by a disease-and cause-agnostic selection gate.
Origin	Nature; arises from biological reality.	Arises from consensus-derived criteria.
Ontological status	Exists independently of investigators	Exists only because investigators define it.
Source of expansion	Scientific discovery.	Administrative or consensus redefinition.
Persistence through time	Generally stable	Changes when enrollment gate changes.
Role of covariates	Variation within one causal system.	Mixture of covariate structures from different causal systems.
Covariate meaning	Variables generally retain a stable interpretation within the disease.	The same variable may have different meanings across diseases.
Source of heterogeneity	Heterogeneity of treatment effects within a disease.	Heterogeneity of treatment effects plus disease-mixture variation.
Treatment-effect estimate	Disease- or mechanism-specific effect.	Mixture-level effect.
Transportability	Potentially transportable to the same disease or mechanism.	Dependent on disease mixture in target population; transport may be harmful
Stability across trials	Expected to remain relatively stable in direction of treatment effect	May vary substantially in direction of treatment effect as disease proportions π_i_ shift across studies.
Primary failure mode	Classical heterogeneity of treatment effects.	Cause-mixture paradox and treatment-effect reversal due to mixture shifts.
Dependence on consensus panels	None. The disease exists whether recognized or not.	Complete. The SDGP exists only because a consensus panel defines the gate.
Research/Grant productivity implication	Constrained, discovery requires identification of genuine causal mechanisms.	Unlimited, new study populations can be generated rapidly through gate redesign without discovery of new biology.

Comparison of Biological Data-Generating Processes (BDGPs) and Synthetic Data-Generating Processes (SDGPs). A BDGP is a coherent biological causal system that exists independently of investigators and can be represented mathematically as a stochastic process. An SDGP is a gate-defined statistical mixture of multiple BDGPs created through disease- and cause-agnostic enrollment criteria. While both are valid statistical data-generating processes, only the BDGP necessarily corresponds to a coherent biological causal system

## Discussion

This structural analysis demonstrates that usual appeal to heterogeneity of treatment effects, as the source of cause-agnostic trial instability is incomplete. In cause-agnostic trials, heterogeneity is not only an inherent property of the population but also can be synthetically shaped by using a disease- and cause-agnostic triage selection gate. The gate, which can be a subgroup gate [36], creates the mixture of causal diseases but also of covariates linked to each of these diseases. Thus the mixture is an aggregation of parallel diseases and covariates as shown in Fig. 2. The resulting trial treatment estimate is a reflection of the instant causal-disease mixture which may never be the same as any other causal disease mixture in another trial or in a clinical population.

Crucially, this disease mixture does not represent merely increased variability. The same observed covariate component may arise from different causal roles across causal systems. A given element of the covariate vector, such as a laboratory value or physiologic measurement, may be a driver of disease severity in one system, a downstream consequence in another, compensation in a third, and causally irrelevant in a forth. Thus, the observed covariate vector is not a single coherent object, but a composite of system-specific covariate structures projected onto a shared covariate framework.

Although reversals in cause-agnostic trials may arise through the cause-mixture paradox, such reversals are merely the visible signal of this deeper structural limitation. More commonly, these CAR designs would be expected to yield null or negative results as divergent disease specific effects cancel each other out so that no statistically significant average result is estimated at the E3 level.

Therefore, generally the core methodological task lies upstream: ensuring that enrollment and intervention are aligned with a coherent causal mechanism. However, in some designs this may be achieved by a downstream physiological gate. Trials that achieve this alignment are more likely to produce transportable knowledge, whereas trials that rely on broad thresholds generate disease mixture-defined estimands require careful interpretation and may introduce risk when translated into clinical guidelines.

Another key discovery from the mathematical analysis is that, in a cause-agnostic trial, the joint covariate–disease mixture is not a stable object of inference. Rather than indexing variation within a single data-generating process, it spans multiple parallel causal systems and conflates within-system variation with between-system composition. While this composition may shift across trials (or within adaptive designs), even within a single trial it prevents covariate-based analyses from mapping to a coherent causal system. Consequently, unless the trial otherwise has a common thread or a funnel somewhere along the path, covariate adjustment, effect modification, and subgroup analysis may be ill-posed from a transport perspective, as they operate on a disease-mixture and covariate-mixture defined estimand rather than a coherent, transportable causal object.

Given the central importance of transportability in trial design even in a standard causal integrity RCT [37], the use of basic structural causal modeling [38, 39], such as directed acyclic graphs (DAGs), during trial development and during review of the trial post hoc, could help investigators assess the degree of causal alignment achieved by the design [40]. This approach would also assist guideline committees in evaluating the evidentiary strength of a trial by clarifying the causal structure underlying the treatment effect estimate (estimand) and by identifying the causal layer (E₂ or E₃) from which the estimand was derived.

The three-estimand framework provides a structural basis for analyzing treatment effect reversals in randomized trials. “Structural treatment effect reversal analysis” (STERA) is defined as an evaluation of whether discordant or reversing trial results can be explained by the use of a cause agnostic gate or pathologies in the underlying trial structure.

Formal structural analysis of past treatment effect reversals is needed given the high clinical stakes of incorrect inference and downstream application. Incorporating STERA into trial evaluation and guideline development has the potential to improve safety, but its implementation will require dedicated funding support.

## Future directions

Future research should focus on developing a general theory of trial gate–to–clinical transport, specifying how different enrollment structures generate distinct estimands and defining the conditions under which those treatment effect estimates remain stable and safely transportable beyond the trial context. Causal inference already provides a theory of transport across populations; what is needed here is a theory that links trial gate design and estimand structure to safe and reliable transport into target clinical populations. This agenda should also include formal delineation of the spectrum and preferred pedagogical boundary between causal-integrity randomized controlled trials (CIRs) and cause-agnostic randomized controlled trials (CARs).

A second area of investigation is the structure of the covariate space in cause-agnostic trials. In particular, the study of temporal and relational time series patterns among covariates in relation to treatment may reveal the coexistence of multiple underlying causal systems, providing a basis for detecting mixture structure, assessing estimand instability, and identifying conditions under which transport is likely to be successful.

Third, social scientists should investigate methods to close the epistemic interface gap between clinical science, causal inference, and statistical practice. Approaches such as “causal symbolic modeling” (cSM) [1], a simplified form of structural causal modeling to biologically explicate symbols, labels, gates, and syndromes, warrant systematic evaluation. The development of simple, easy to learn, standardized causal diagrams and formal frameworks for assessing causal coherence should be pursued and incorporated into grant applications, peer review, and guideline development as clinicians have little time to engage the complexity of causal inference as a field. The accumulated record suggests that the separation of these domains within clinical science contributes to persistent epistemic interface gaps and this has not served the public health well in critical care science.

Finally, the scientific community should investigate the history, nature, and sources of synthetic data-generating processes (SDGPs) and develop formal tools to identify and distinguish them from coherent causal systems. Frameworks such as STERA and causal symbolic modeling (cSM) provide a basis for this effort and warrant systematic evaluation. These approaches would enable prospective assessment of trial design and transport validity and should be integrated into funding criteria, peer review, and guideline development, with the aim of eliminating the routine creation and study of SDGPs. SDGP-based investigations constitute a form of unlimited “disposable research” consuming resources while generating potentially harmful results. These should be displaced from the funding stream in favor of designs that have been assessed to optimize the generation of durable, mechanism-aligned knowledge, regardless of study outcome.

Empirical work is also needed to apply these concepts to existing trials and guideline recommendations. In parallel, prospective monitoring systems, analogous to real time failure mode and effects analysis, should be developed to detect early signals of harm when trial-derived protocols are deployed at scale.

One example illustrating the importance of distinguishing SDGPs from BDGPs is the widespread use of trial enrichment strategies, defined as the prospective use of patient characteristics to select a study population in which detection of a treatment effect is more likely [41]. However, the proper application of enrichment requires recognition of the underlying RCT framework. Predictive enrichment generally tends to improve BDGP alignment because it selects patients on the basis of characteristics linked to the treatment mechanism. In contrast, prognostic enrichment requires proper alignment in the first instance. A stand-alone prognostic gate, whether enriched or not, may continue to generate an SDGP by selecting a heterogeneous mixture of distinct biological data-generating processes. As a result, the apparent increase in treatment effect may reflect selection within a disease mixture which is still subject to the cause mixture paradox, rather than improved alignment with a coherent causal mechanism.

Finally, these mathematical findings have implications for research governance. Incorporating structural causal modeling into funding, trial registration, and reporting standards may help ensure that randomized trials are designed around coherent causal targets. Without such reforms, continued consensus generation and amendment of SDGPs, and reliance on syndrome-based, cause-agnostic trial designs, risks generating internally valid but non-transportable evidence, with potential consequences for patient safety when translated into clinical practice.

## Conclusion

This mathematical analysis demonstrates that critical care syndrome RCT with consensus derived cause-agnostic enrollment criteria do not merely average over patient-layer treatment effects and covariate variation within a single disease or causal mechanism. Instead, they introduce an additional structural layer (E₃) that generates a second order average, that is, an average of disease-specific average treatment effects (E₂). This second-order average is a mixture-weighted average across fundamentally different diseases and causal mechanisms, each with its own covariate distribution. Without formal mathematical and gate analysis, this second-order average treatment effect can be readily misconstrued as possessing the high evidentiary strength traditionally associated with a well-designed randomized controlled trial. The mathematical structure presented here demonstrates that this form of “evidence-overconfidence error” is a predictable and potentially correctable source of RCT instability and subsequent guideline reversal.

This mathematical analysis further indicates that, in the design of clinical trials, equal priority should be given to gate associated structured causal alignment, transportability, and statistical precision [37]. Achieving this will require the development of a formal gate–estimand–transport theory. This is required because, even when causal alignment is achieved at the trial level, transportability to individual patients remains a separate challenge requiring careful consideration by guideline committees and clinicians [37].

This structural analysis does not suggest that all cause-agnostic trials are invalid, but that their treatment effect estimates differ structurally from disease or mechanism specific trials and therefore require different interpretation when used for guideline development. The three estimand structure derived mathematically herein clearly demonstrates that one key issue for a guideline committee in evaluating the “level of evidence” of a trial is to determine whether the estimand is at Layer 2 or Layer 3 and then if it is Layer 3, is there sufficient physiologic funneling.

## Data Availability

No datasets were generated or analysed during the current study.

## Abbreviations

AKI: Acute Kidney Injury
ARDS: Acute Respiratory Distress Syndrome
ATS: American Thoracic Society
BDGP: Biological Data–Generating Process
CAP: Community–Acquired Pneumonia
CAR: Cause–Agnostic Randomized Controlled Trial
CcO₂: End–Pulmonary Capillary Oxygen Content
CaO₂: Arterial Oxygen Content
CIR: Cause–Integrity Randomized Controlled Trial
CRT: Capillary Refill Time
cSM: Causal Symbolic Modeling
CvO₂: Mixed Venous Oxygen Content
DAG: Directed Acyclic Graph
DGP: Data–Generating Process
E₁: Individual–Level Treatment Effect
E₂: Disease–Specific Average Treatment Effect
E₃: Disease–Mixture Average Treatment Effect
FiO₂: Fraction of Inspired Oxygen
ICH: International Council for Harmonisation of Technical Requirements for Pharmaceuticals for Human Use
PEEP: Positive End–Expiratory Pressure
PaO₂: Arterial Partial Pressure of Oxygen
P/F Ratio: PaO₂/FiO₂ Ratio
Qs/Qt: Intrapulmonary Shunt Fraction
RCT: Randomized Controlled Trial
SDGP: Synthetic Data–Generating Process
SIRS: Systemic Inflammatory Response Syndrome
SOFA: Sequential Organ Failure Assessment
SSC: Surviving Sepsis Campaign
STERA: Structural Treatment Effects Reversal Analysis
SvO_2_: Mixed Venous Oxygen Saturation

## Glossary

BDGP: Biological Data-Generating Process. A coherent biological causal system existing independently of investigators
CAR: Cause-Agnostic Randomized Controlled Trial. An RCT whose enrollment gate is defined by disease- and cause-agnostic clinical criteria rather than a single biological mechanism
Cause-mixture paradox: Reversal of observed trial effects caused by changes in the underlying disease mixture despite stable disease-specific treatment effects
Cause-mixture heterogeneity: Variation arising from differences in disease composition across enrolled patients rather than variation within one disease
CIR: Cause-Integrity Randomized Controlled Trial. An RCT whose enrollment gate selects one coherent disease or causal mechanism
Covariate heterogeneity: Variation among patients within a single disease or causal mechanism, represented mathematically by P(xi ∣ D = i, S = 1)
Disease mixture: The proportions of different diseases or causal mechanism included by the enrollment gate, represented by πi(S = 1)
E₁: Individual patient treatment effect within one disease
E₂: Disease-specific average treatment effect estimated within a CIR
E₃: Mixture-weighted average of disease-specific treatment effect estimated within a CAR
Estimand: The mathematical treatment-effect object generated by a trial's structural design. In this framework, the enrollment gate determines whether the estimand represents a disease-specific effect (E₂) or a disease-mixture effect (E₃)
Estimand Layer Error: Misinterpreting an E₃ disease-mixture estimand as if it were an E₂ disease-specific estimand
Gate–Estimand–Transport Theory: Proposed framework linking enrollment structure, estimand, and transportability
HTE: Heterogeneity of treatment effects occurring within a coherent causal system
Mixture weights (π_i_): The proportions of each disease among enrolled participants
P(x_i_ | D=i, S=1): Joint covariate distribution within one disease after enrollment
SDGP: Synthetic Data-Generating Process. A trial-defined mixture of multiple biological causal systems produced by a disease- and cause-agnostic enrollment gate
STERA: Structural Treatment Effects Reversal Analysis. A framework for determining whether discordant trial results arise from differences in trial structure rather than biology
Transportability: The ability to validly apply a trial’s selection gate and its resulting treatment-effect estimate to a target population outside the original trial
Triage selection gate: The consensus based mechanism creating the enrolled disease mixture
